# “CATCH” Study: Correct Antibiotic Therapy in Continuous Hemofiltration in the Critically Ill in Continuous Renal Replacement Therapy: A Prospective Observational Study

**DOI:** 10.3390/antibiotics11121811

**Published:** 2022-12-13

**Authors:** Alberto Corona, Alice Veronese, Silvia Santini, Dario Cattaneo

**Affiliations:** 1 Accident & Emergency and Anaesthesia and Intensive Care Medicine Department, Esine and Edolo Hospitals, ASST Valcamonica, 25040 Brescia, Italy; 2 Intensive Care Unit, ASST Fatebenefratelli Sacco, Polo Universitario, Via GB Grassi 74, PO Luigi Sacco, 20157 Milano, Italy; 3 Intensive Care Unit, ASST Ovest Milanese, Via Giovanni Paolo II, 20025 Legnano, Italy; 4 Unit of Clinical Pharmacology, ASST Fatebenefratelli Sacco University Hospital, Via GB Grassi 74, 20157 Milan, Italy

**Keywords:** pharmacodinamic, parmacokinentics, antibiotic, acute kidney failure, critically ill patients, CVVH

## Abstract

The proper posology of antibiotics in the critically ill in CRRT is difficult to assess. We therefore performed a prospective observational cohort study to make clear hints in this topic. Our results reveal a high Sieving Coefficient for all antibiotics, equal to or higher than those described in previous papers. CVVH clearance in relation to total body clearance was significant, (i.e., >than 25% for all classes). A strong correlation between the antibiotic concentrations obtained in plasma and ultrafiltrate was found both at the peak and in the valley, with the determination of two equations that allow a new method for calculating the amount of antibiotic lost in CVVH both for trough levels and peak. Based on the results of our study and considering the limitations we believe that we can extrapolate the following final considerations: (1) it is likely to carry out a loading dose for the main antibiotics (2) subsequent administrations must take into account the daily loss identified by the linear regression equation. This angular coefficient gives the idea that the average daily loss of given antibiotic is about 25%; this implies that on the basis of the linear regression equation that correlates ultrafiltered/plasma antibiotic concentration, the dosage should be increased by 25% every day, while still ensuring a daily plasma TDM of the drug.

## 1. Introduction

Acute kidney failure (AKI) is a condition easily encountered in intensive care unit (ICU), associated with a high mortality rate (30–35%) particularly in case of occurrence of sepsis. The indications of treating AKI with Continuous Renal Replacement Therapy (CRRT) is increasing, since it is supposed to ensure a better outcome the earlier it is commenced. Furthermore optimal and adequate antibiotic therapy is assessed to impact on critically ill experiencing sepsis in AKI, however the drug elimination rate during CRRT can be highly variable, depending both on the method used and on the characteristics of the patients [1,2].

Sepsis itself is used to increase drug volume of distribution (V_d_), and to prolong its half life (T_1/2_) and to alter the protein binding of many antibiotics inducing an increased capillary permeability due to the release of inflammatory mediators causing eventually fluid accumulation and hypoalbuminemia [3,4,5,6]. The parameters set on the machine can modify clearance in various ways: the increase in blood flow or dialysate can affect the cartridge transmembrane pressure and therefore the removal of antibiotics [7]. The removal of drugs in the critically ill patient in CRRT is affected by pharmacokinetic factors and could be related to type, dose and profile of prescriptions. Although the KDIGO guidelines recommend an effluent dose of 20–25 mL/kg/ h it has been found that the initial prescribed dose is often higher [3,8] and therefore increasing the risk of being sub-therapeutic at the standard dosage of antibiotics [7,9]. Due to the lack of defined guidelines on the dosage of antibiotics in CRRT, many models based on equations, often complicated and not supported by clinical data [10,11,12,13,14], have been proposed, but only on unproven theoretical assumptions.

As a consequence of these assumptions, it may therefore be necessary to increase the doses compared to the patient in renal insufficiency without CRRT therapy, although we tend to consider the patients in CRRT with a reduced renal clearance [15]. It is clear that it is not possible to identify a standard dosage, because there is a risk of oversimplification, failing the therapeutic goal. Indeed, it has been seen that in 25% of cases the antibiotic therapy does not reach the necessary plasma concentration target, due to lack of achievement of the MIC (15%) considered optimal and bactericidal [16].

In this context, our study, whose acronym is CATCH (Correct Antibiotic Therapy in Continuous Hemofiltration) was conceived and conducted with the aim of informing clear hints in the controversial topic of antimicrobial posology in critically ill patients in AKI and CRRT.

## 2. Results

### 2.1. Demographic Characteristics of the Studied Population

During the study period, 40 patients were recruited, whose demographic characteristics are summarized in the following Table 1. Particularly high is the severity of illness as highlighted by the SMR and the mortality related to admission value of the SOFA score. Indeed, more than half the patients were in severe renal failure as indicated by RIFLE and AKIN criteria (see Appendix A).

Table 2 shows the type of antibiotics whose plasma and ultrafiltrate TDM was determined and number of determinations.

### 2.2. Pharmacokinetic Curves and Measured Parameters

Only the data related to the antibiotics of which there is more than 5 determinations will be presented. The dosages of the antibiotics were prescribed according to the indications of the “Sanford antimicrobial guide” (2018 Edition) and subsequently modified according to the TDM.

**Figure 1:**
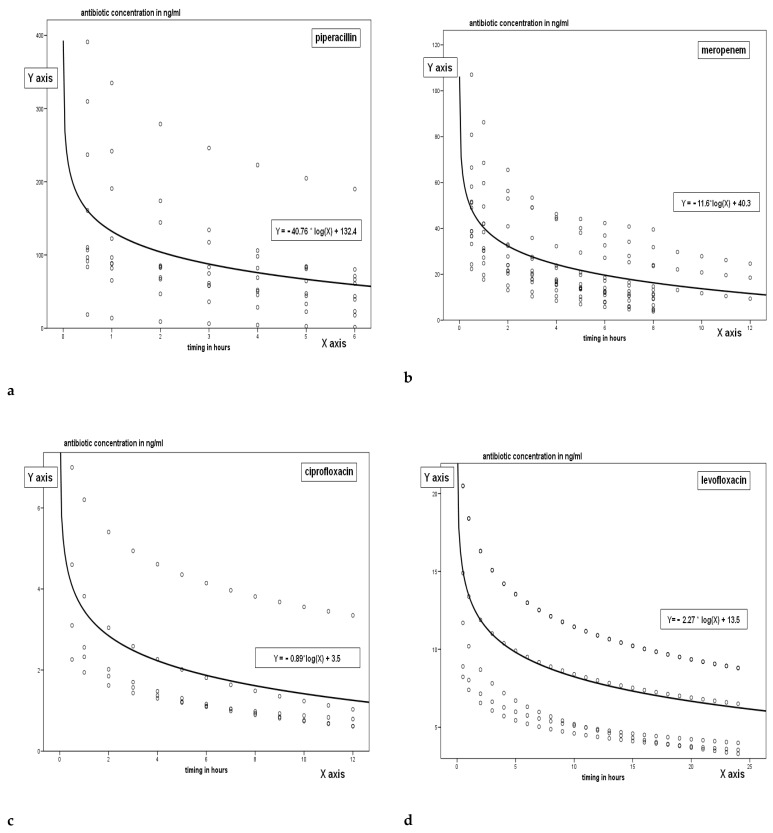

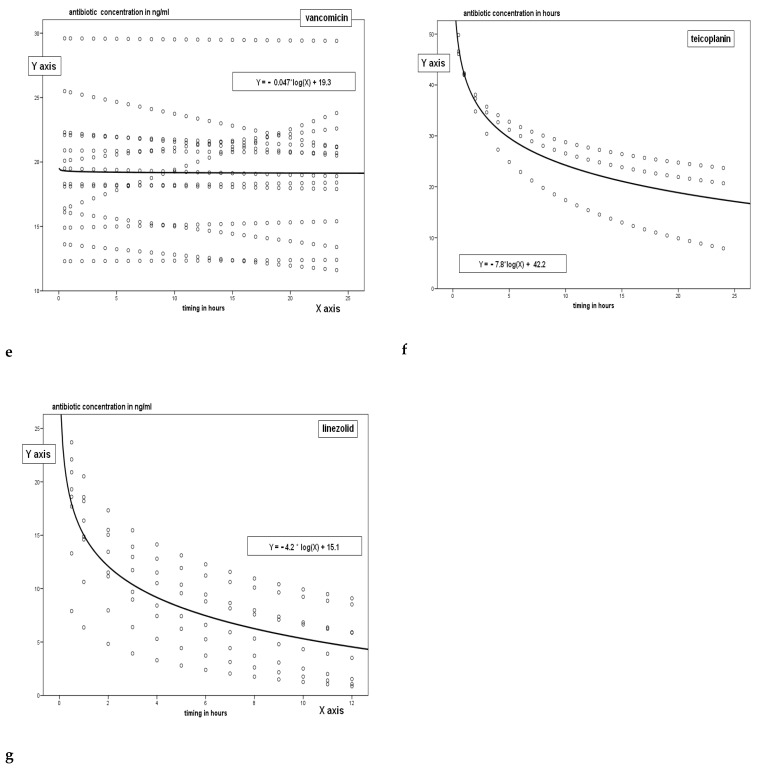
Pharmacokinetics curves for each tested.

Figure 1a–g shows the pharmacokinetic curves obtained for each class of antibiotic; the trend is related to time and particularly since the peak concentration, measured after half an hour since the drug administration up to the time immediately before the following administration of the antimicrobial. The trend of the single antimicrobial concentrations can be interpreted by a logarithmic regression curve whose specific equation is shown in the figures.

The only drug with different concentration trend in vancomycin, since given in continuous infusion.

The dots shown in the figures represent the PK trend of concentration of the drug in relation to time; trend is extrapolated between peak concentration and trough level with the rational hypothesis of a logarithmic decay of the concentration. The line represents the logarithmic regression curve which is the interpretative synthesis of all the TDM both measured and extrapolated.

Table 3 shows PK parameters. We should like highlight that Ke^−1h^: epresenting the elimination rate coefficient of the drug at the first hour-[define as the ratio of CL_tot_ (L/h)/V_d_ (L/Kg)] is higher in the drugs with lower molecular weight and reduced steric hindrance (piperacillin, meropenem, ciprofloxacin). V_d_ (L/Kg) present a wide variation and their values are higher than recorded ones in the literature. A statistical significance has been recorded comparing the variation of AUC (*p* = 0.001), Ke^−1h^ (*p* = 0.001), CL_tot_ L/h (*p* = 0.001), CL_CVVH_ ml/min (*p* = 0.001), V_d_ L/Kg (*p* = 0.001), using ANOVA test (with post hoc correction of Bonferroni).

### 2.3. Sieving Coefficients and Evaluation of Clearance in CVVH

**Figure 2:**
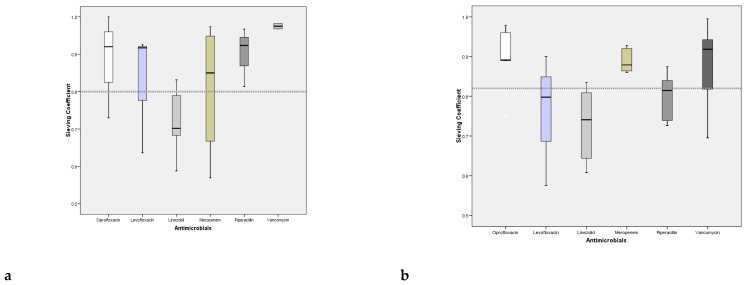
Sieving coefficients at the peak and valley of the antibiotics studied (continuous infusion for Vancomycin has been used).

Figure 2a,b represent the Sieving Coefficient for each antimicrobial derived from the ratio between TDM of the drugs in UF and plasma; a wide variation has been recorded both in trough levels values and in peak ones (*p* = 0.01, ANOVA for both trough levels and peak).

**Figure 3:**
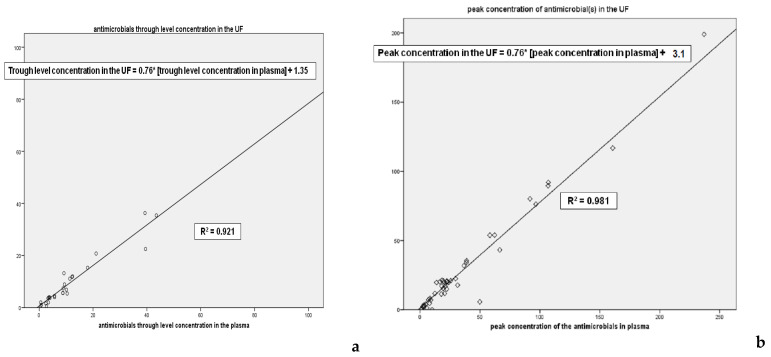
Lines and regression coefficient of the concentration of antibiotics in plasma in UF at the trough levels and peak concentration. * Multiplication sign.

Figure 3a,b show the linear regression between the concentration of the drug in UF and plasma; values are presented for both trough level and peak concentration; the relation between the drug concentrations in the two fluids is very close, confirmed by the R2 presented in the figures. The higher is the plasma concentration of the antimicrobials the higher is the UF one.

Moreover the angular coefficient of the two lines is 0.76 allowing the demonstration that the antimicrobial concentration of UF is lower of 25% of plasma one, since such a quantity of drug is removed (complementary and integrating the considerations about the SC, see Figure 2a,b).

**Figure 4:**
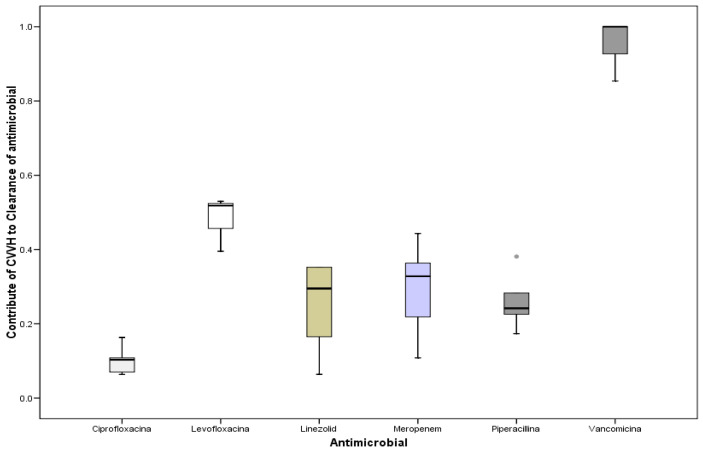
Contribution of antibiotic clearance linked to CVVH in relation with the total body clearance.

Figure 4 shows the contribution of antibiotic clearance linked to CVVH in relation with the total body clearance; a wide variation has been recorded (*p* = 0.001, ANOVA)

CVVH-related clearance was obtained with the following formula:

$${\mathrm{CLcvvh}}_{\mathrm{ATB}}=C_{s}*\left[Q_{uf}*\left(\frac{Q_{p}}{Q_{p}+PBP}\right)\right]$$

*C_s_* = Sieving coefficient

*Q_uf_* = effluent flow

*Q_p_* = plasma flow

*PBP* = blood pre-pump flow

As highlighted in the following Table 4, the lost of antibiotics in the ultrafiltrate for all six antibiotic.

## 3. Discussion

The optimal dosage of antibiotics in the critically ill patients is difficult due to the numerous variables that can affect the Pk and Pd of the drug, such as alterations in V_d_, protein binding and body clearance, due to impairment of the perfusion and organ dysfunction and/or failure. It is even more difficult in the case of CRRT, both for the number of techniques available and for the possible different settings. Clinical studies are at the moment enough limited to inform practice in this topic.

The treatment of ARF can be based on the inhibition of the mechanism of renal damage, on the enhancement of the recovery process from the insult suffered and on a replacement, support and control of complications. In the case of the critically ills, CRRT is adopted since it is the most appropriate one in the management of patients with haemodynamic instability. Often the parameters of the CRRT are not clearly specified or different methods are considered together. Furthermore, some Pk/Pd parameters are deduced from healthy volunteers or obtained from formulas without direct measurements [5,7,9,10,15,16,17,18,19]. If the advantages are constituted by the continuous removal of toxins, a greater hemodynamic stability, an easier control of the water balance and no increase in intracranial pressure induced by the treatment, on the other hand there is a consensual removal of drugs and therefore of antibiotics, especially in the case of the use of specific cartridges (i.e., OXiris/Septex). The prevalence of sepsis in the critically ill is 51% and 70% of hospitalized patients receive antibiotics and the infection rate increases from 30 to 70% between the first and seventh day of ICU stay. The main risk factors for infection undoubtedly include acute renal failure and the need for RRT. In addition, infected patients appear to have more comorbidities and clinical conditions of greater severity upon admission than non-infected, with higher SOFA and SAPSII scores. The main sites of infection were the respiratory system (64%), the abdomen (20%), the circulatory system (15%) and the genitourinary tract (14%), with microbiological isolations in 70% of the cases. Among the isolated pathogens there is a strong increase in gram-negatives (62% of isolates compared to 50% of previous studies [20,21], against 47% of Gram-positive, while anaerobes amounted to 5%. From what has been said previously, the optimal use of antibiotics is fundamental for the frequency and impact that the infectious disease has on the outcome in the critically ill patient, also given the increase in microbial resistance [22,23,24]. However, 30 to 60% of prescriptions have been found to be inappropriate or sub-optimal [22,25].

Pharmacokinetics (Pk) describes the absorption, distribution, metabolism and elimination of the drug. These variables determine the concentration obtained in the circulation and, consequently, in the tissues, and its variation over time. Important parameters are:

- bioavailability, i.e., the amount of drug absorbed into the systemic circulation after administration (100% for the intravenous route)
- the volume of distribution (V_d_), i.e., a virtual volume in which the total amount of a drug present in the body should be uniformly distributed to obtain the same concentration measured in the plasma [26,27]. Hydrophilic drugs will have a reduced V_d_, limited to the bloodstream, while lipophilic drugs will tend to accumulate in the body for penetration into cells and adipose tissue, with a high V_d_. In turn, this will affect the minimum (C_min_) and maximum (C_max_) concentrations obtained in the circulation after one administration and the time to reach them (T_max_−T_1/2_). It is an indispensable parameter for establishing the initial dose of administration of a drug [26,27,28], given by the product of V_d_ for the desired plasma concentrations [C] and for the body weight D = C × V_d_ × body weight
- drug clearance, i.e., the volume of blood purified in a given time interval.
- The half-life of a drug (T_1/2_), i.e., the half-life of the concentrations reached, which is important for defining the interval between administrations. It is closely linked to clearance (Cl) and V_d_ [26]: T_1/2_ = 0.693 × V_d_/Cl
- the protein binding, that is, the portion of the drug linked mainly to albumin, the free portion being the one that carries out the pharmacological activity.
- the area under the concentration-time curve (AUC), which reflects the exposure of tissues to the drug over time.

In the critically ill patient there are numerous modifications that can alter the Pk of drugs and in particular of antibiotics [29]. First of all, the methods of administration other than the intravenous one are not reliable, due to the possible presence of edema or peripheral hypoperfusion that make the effect unpredictable if used the subcutaneous/intramuscular route and due to changes in intestinal absorption due to alterations in gastric pH, peristalsis, of perfusion or by exclusion of portions of the intestine for blind loops or ostomies.

The V_d_ appears to be increased in the majority of critically ill patients, due to dyscrasia with a tendency to the third space and due to fluidic overload. Consequently, for a given drug dose, the C_max_ will be lower on the one hand but T_1/2_ will increase, with a possible tendency to accumulation. The amount of drug bound to proteins also changes, because on the one hand there is a tendency to hypoalbuminemia, with less binding especially for acid drugs, and on the other hand an increase in alpha1-glycoproteins related to inflammation, with greater binding basic drugs. Finally, due to the presence of organ dysfunction, with possible alteration of liver and kidney function and therefore a reduction in clearance. All this affects the Pd of the antibiotic, or the relationship between its mechanism of action and the concentration reached. Time and concentration-dependent drugs can be identified [25,26,27,28,29]. For the former, the effectiveness of bacterial elimination is linked to the time during which their concentration in circulation is maintained above the minimum concentration that in vitro prevents bacterial replication (MIC) (T > MIC, with a concentration of 4–5 times the MIC). For this type, continuous infusion or prolonged infusion at each administration is indicated, with at least 50% of the time above the MIC with an ideal of 100%. For the latter, on the other hand, it is linked to the peak concentration reached with respect to the MIC, with a target of 8–10 times (C_max_ > MIC or AUC/MIC). A single administration is therefore more useful. Some antibiotics also exhibit a post-antibiotic effect (PAE), i.e., the ability to suppress bacterial growth even when the concentration falls below the MIC [29,30,31,32].

The need for CRRT is increasing, also because it ensures a better outcome the earlier it is applied. Optimal antibiotic therapy is essential in these patients, but the drug elimination rate during CRRT can be highly variable, depending on the method used, the characteristics of the same and the patient’s condition. Sepsis itself increases V_d_, prolongs t_1/2_ and alters the protein binding of many antibiotics, with increased capillary permeability due to the release of inflammatory mediators with fluid accumulation and hypoalbuminemia [3,4]. For example, the V_d_ of aminoglycosides increases by about 25% in the critically ill patient, while the V_d_ of beta-lactams and of Vancomycin changes less but with important individual variations [5,6].

Antibiotics with low volume of distribution (<1 L/kg) will be more affected by removal during CRRT than those with high V_d_ (>2 L/kg), especially in the course of slow removal techniques, i.e., the main ones used in intensive care, due to the possibility of a continuous redistribution of the drug from the tissues to the blood [5]. The parameters set on the machine can modify clearance in various ways: the increase in blood flow or dialysate can increase the transmembrane pressure and therefore the removal of antibiotics [7]. Although the KDIGO guidelines recommend an effluent dose of 20–25 mL/kg/h it has been found that the initial prescribed dose is often higher [3,8]. With this the risk of being sub-therapeutic at the standard dosage of antibiotic, with the need for higher dosages [7,9]. The choice to replace the fluids removed in pre-dilution or in post-dilution will have an impact on drug clearance, reducing it in the first case and enhancing it in the second [17]. Furthermore, the use of biosynthetic membranes, with larger pores than conventional cartridges (i.e., Oxiris and Septex), involves the removal of drugs with a higher molecular weight. [5,7,9,33] Finally, the duration of use of the circuit may also have a role in the removal of drugs. In fact, there is a tendency for the formation of a second membrane on the filter for protein deposition from the plasma during CRRT, with a reduction in transmembrane clearance and therefore in the performance of the filter [33,34].

There are factors related to (1) PKs: (i) residual renal function; (ii) non-renal clearance (iii) V_d_ (if increased, there is less efficacy of clearance in CRRT); (iv) protein binding; and (2) related to CRRT; (i) type and prescribed dose of CRRT; (ii) blood flow; (iv) cartridge type surface.

Due to the lack of defined guidelines on dosing of antibiotics in CRRT, many equation-based models, often complicated and unsupported by clinical data, have been proposed, as shown in Table 5 [10,14]. In fact, the clearance of CRRT is significant when this represents more than 25–30% of the total body clearance for a given drug, but for many of them the clearance is estimated and not measured [5]. Furthermore, the Sieving coefficient, which indicates how permeable the cartridge membrane is to the drug, is often calculated based on the protein binding of the drug, obtained from tables based on data in healthy volunteers, not reflecting the conditions present in the critically ill patient [5,17].

The clearance of CRRT is considered relevant for drugs with predominantly renal elimination, with a reduced V_d_ and low protein binding, and therefore a significant Sieving coefficient [17]. As for antibiotics, therefore, it should be hydrophilic in particular that should be concerned. However, there are exceptions: for example, among the Beta-lactams, Ceftriaxone and Oxacillin have mainly biliary elimination; among the Fluoroquinolones, Levofloxacin and Ciprofloxacin, despite their lipophilicity, have renal clearance [15]. Therefore, it may be necessary to increase the doses compared to the patient in renal insufficiency without replacement therapy, although we tend to consider the patient in CRRT with a reduced clearance, considering the GFR between 10 and 30 mL/min [15]. From this it is clear that it is not possible to identify a standard dosage on this assumption, because there is a risk of oversimplification, failing the therapeutic goal [16].

Given these premises, the advantages of this clinical study are the following: 

(1) Our results reveal a high Cs for all antibiotics, equal to or higher than those described in previous papers [15,26]. This is predictable given the type of drugs studied, with reduced V_d_, as known and confirmed by us [5,25,26,29]. In fact, CVVH clearance in relation to total body clearance was significant, i.e., greater than 25% for all classes [5]. This may suggest that a reduced dosage for these antibiotics is not mandatory, taking into account the risk of being subtherapeutic and developing microbial resistance.

(2) We also highlighted a strong correlation between the antibiotic concentrations obtained in plasma and ultrafiltrate, both at the peak and in the valley, with the determination of two equations that allow a new method for calculating the Cs, starting from a single dosage plasmatic from which it is possible to derive that of the ultrafiltrate, and therefore their ratio (Figure 3a,b).

- [C] ATB (UF) Peak = 0.76 × [C] ATB (plasma) + 3.1
- [C] ATB (UF) trough level = 0.77 * [C] ATB (plasma) + 0.93

(3) Furthermore, by obtaining the concentrations in the ultrafiltrate, it is possible to estimate the drug loss with CVVH in mg/h with the following formula:

- [C] ATB loss = CVVH dose * [C] ATB (UF)

Being: [C] loss = mg/h of drug lost with the ultrafiltrate, CVVH dose = by convention in our study 25 mL/kg/h, [C] ultr = drug concentration in the ultrafiltrate in mg/L, which can be obtained by applying the previous equations without the need for dosage.

The main limitations are the following one:

(1) the observational nature of the study and the reduced number of the sample size, although the epidemiological unit is the single antibiotic TDM
(2) the choice of a specific method of CRRT, the CVVH in exclusive haemofiltration in citrate, with a standard dose of 25 mL/kg/h, if make easier the determination of Cs, do not allow to extrapolate a general practice for any kind of CRRT.
(3) the selectivity on the anuric patient allows to eliminate another variable, diuresis, and therefore any residual renal clearance; unfortunately this is one of inclusion criteria that does not help to increase sample size.
(4) As regards the type of antibiotics examined, they appear to be those mainly investigated in the literature because of widespread diffusion. In our series, cephalosporins are not available, due to the impossibility of TDM, and aminoglycosides due to our scarce use. Therefore we limited only a 6 antibiotics, excluding the newer; however our intention is to carry on the study in the future with higher sample size and enlarging number of studied antibiotics,

## 4. Methods

### 4.1. Type of Study

Cohort prospective observational clinical study conducted at the Multipurpose Resuscitation Department of the Luigi Sacco Hospital in Milan, from January 2017 to January 2020, interrupted due to the onset of the Pandemic due to COVID 2019.

### 4.2. Objectives of the Study

This study aims to evaluate the ongoing removal of CVVH of the most common antimicrobial drugs used in our resuscitation and to detect the PK/PD parameters in the critically ill patient with AKI.

### 4.3. Inclusion Criteria

- Totally anuric patient (less than 100 mL/24 h, in a view to definitely reduce the residual clearance of the patient)
- Antibiotic therapy with at least one of the antibiotics under study, of which TDM (Therapeutic Drug Monitoring) is available
- Continuous renal replacement treatment in exclusive ultrafiltration (CVVH) with reinfused in post dilution to reach 25 mL/Kg/h of CVVH dose

### 4.4. Exclusion Criteria

- Residual diuresis (>100 mL of urinary output in 24 h)
- Expected RRT duration less than 48 h
- Non-convective mode in exclusive ultrafiltration (CVVH) with reinfused in post dilution to reach 25 mL/Kg/h of CVVH dose
- Age < 18 years

### 4.5. CVVH Profile

- Prismaflex^®^ SW 5.XX system set
- M150 hemofilter
- Anticoagulation in citrate
- Forced pre-dilution in relation to pump speed
- Reinfused in post dilution to reach 25 mL/Kg/h of CVVH dose

### 4.6. Timing of Sampling

- At steady state, by convention on the third day
- On serum and ultrafiltered
- Single-dose antibiotics
- One administration/24 h
- In 30 min
- Dosages on ultrafiltrate and serum 30 min after the end of the administration 24 h later, just before the next one

### 4.7. Double Administration Antibiotics

- Two administrations/24 h (one every 12 h)
- In an hour
- Dosages on ultrafiltrate and serum 30 min after the end of the administration 12 h later, just before the next one

### 4.8. Triple Administration Antibiotics

1. Three administrations/24 h (one every 8 h)
2. In an hour
3. Dosages on ultrafiltrate and serum 30 min after the end of the administration 8 h later, just before the next one

### 4.9. Quadruple Administration Antibiotics

- Four administrations/24 h (one every 6 h)
- In 30 min
- Dosages on ultrafiltrate and serum
- 30 min after the end of the administration
- 6 h later, just before the next one

### 4.10. Continuous Infusion Antibiotics

- Loading dose in one hour
- Dosages on ultrafiltrate and serum at least 24 h after charging

### 4.11. Methods of Analysis of Samples

The determination of the concentrations of Ciprofloxacin, Levofloxacin, and Caspofungin was performed with high performance liquid chromatography techniques with mass spectrometry (LC-MS/MS). Before injection into the chromatograph, the biological samples were subjected to centrifugation (3000× *g* 10 min); the derived supernatants were added with trifluoroacetic acid for protein precipitation, centrifuged again (10,000× *g* for 10 min), transferred into microvials and processed. The compounds to be analyzed were separated using reverse phase chromatography columns (C8/C18) and mobile phases composed of variable quantities (gradients) of organic (methanol / acetonitrile) and inorganic (aqueous buffers of acetate salts) phases. The determination of the concentrations of Linezolid, Meropenem, Piperacillin, Voriconazole, Rifampicin, Sulfamethoxazole and Trimethoprim was performed with high performance liquid chromatography techniques with ultraviolet detector (HPLC-UV). Before injection into the chromatograph, biological samples were centrifuged (3000× *g* 10 min); the derived supernatants were spiked with a precipitating agent (perchloric acid for Linezolid, Sulfamethoxazole and Trimethoprim; methanol for Piperacillin, Meropenem, Rifampicin and Voriconazole), centrifuged again (10,000× *g* for 10 min), transferred into microvials and processed). The compounds to be analyzed were separated using reverse phase chromatography columns (C8/C18) and mobile phases composed of variable quantities (gradients) of organic (methanol/acetonitrile) and inorganic (aqueous buffers of acetate salts) phases), with the sole exception of Linezolid for which isocratic conditions were used.

A specific internal standard was used for each analyte processed with chromatographic techniques, and calibration curves were constructed by correlating the known concentrations with the ratio between the height of the chromatographic peak of the drug to be analyzed with the height of the chromatographic peak of the internal standard.

The equations derived from the calibration curves were used for the quantification of unknown samples (also added with internal standard). The determination of the concentrations of Vancomycin and Voriconazole is based on a homogeneous phase immunoenzymatic analysis technique. The determination is based on the competition between the drug in the sample and the one labeled with the enzyme glucose-6-phosphate dehydrogenase (G6PDH) for the binding sites of the antibody.

The activity of the enzyme decreases following the binding with the antibody, and this allows to measure the concentration of the drug in the sample in terms of enzymatic activity. The active enzyme converts oxidized nicotinamide adenine dinucleotide (NAD) into NADH, causing a change in absorbance which is measured by spectrophotometry. The endogenous G6PDH of the serum does not interfere as the coenzyme works only with the bacterial enzyme used in the analysis The performance of all the bioanalytical methods used was monitored: (a) during each analytical run through the processing of internal quality controls and (b) periodically through the processing of external quality controls.

Parameters evaluated for each antibiotic:

Construction of pharmacokinetic curves with detection of:

- AUC
- See
- Ke
- Sieving coefficient
- Clearance in CVVH
- Total body clearance

For each patient the following were detected:

- Severity score
- SOFA and SAPSII
- AKI evaluation criteria
- RIFLE criteria
- AKIN criteria
- Demographic characteristics
- Values of albuminemia

### 4.12. Statistical Analysis

SPSS software (IBM SPSS statistics) version 26.1 was used for statistical analysis. The statistical significance was considered for values of *p* < 0.05 (error α) whereas the power of the statistical tests equal or higher than 95% (error β). The quantitative variables were compared using the Student’s *t*-test, while the non-parametric Mann-Whitney test was applied in case of non-normal distribution of the values. Differences in proportions were determined by the use of the Χ^2^ test with the Fischer’s correction in case of expected values lower than 5. Linear regression was used to compare plasma and ultrafiltrate antibiotic concentration values. Logarithmic regression was use to study the concentration trend of each antibiotic in relation to time.

## 5. Conclusions

Based on the results of our study and considering the limitations of limited sample size and methodology limited to CVVH only and a small number of antimicrobials, although they are the most frequently used, we believe that we can extrapolate from the linear regressions obtained the following final considerations:

- it is likely to carry out a loading dose for the main antibiotics whether they are administered in continuous infusion or bis/ter/quater in die that corresponds to double the standard dose (for vancomycin we recommend 1 gr).
- subsequent administrations must take into account the daily loss identified by the linear regression equation.
- these linear regression lines have an angular coefficient equal to 0.76 and 0.77 respectively for peak and valley which relate the drug concentration on plasma and on ultrafiltrate.
- this angular coefficient gives the idea that the average daily loss of given antibiotic is about 25%; this implies that on the basis of the linear regression equation that correlates ultrafiltered/plasma antibiotic concentration, the dosage should be increased by 25% every day, while still ensuring a daily plasma TDM of the drug.

## Figures and Tables

**Table 1:** Demographic characteristics of the studied population.

Quantitative Variables	Mean (SD)	Median (IQR)
Male: Female ratio	2.45:1	/
Age	64.6 (12.7)	62 (48–72)
Height (cm)	170.8 (8.72)	172 (166–178)
Weight (Kg)	80.6 (21.8)	82 (68–101)
SAPS II	52.9 (15.8)	54 (38–64)
SMR (§) (95% CI)	0.53 (0.19–0.82)	
SOFA	10.4 (3.5)	10 (8–12)
Expected mortality related to SOFA (95% CI)	45% (25–55)	
Albuminemia mg/dl	2428.6 (506.1)	2510 (2280–2910)
Categorical Variables for AKI	Patients #	Patients %
RIFLE (*) 1(R) 2(I) 3(F) 4(L) 5(E)	2 5 24 3 6	5 12.5 60 7.5 15
AKIN (*) 1 2 3	4 4 32	10 10 80

Legend: § = Standardised Mortality Ratio; * see in the Appendix A of the manuscript). The most frequent recorded infections were: peritonitis (18 pts), followed by community acquired pneumonia (8 pts), bacteraemia (6 pts) urinary tract infections (5 pts) and myocarditis, mediastinitis, fever of unknown origin (1 pts for each).

**Table 2:** Dosed antibiotics and number of determinations.

Antimicrobials	Determinations
Rifampicin	1
Teicoplanin	5
Ciprofloxacin	8
Levofloxacin	7
Linezolid	9
Piperacillin/Tazobactam *	9
Meropenem	17
Vancomycin	18
Trimethoprim	1
Sulfamethoxazole	1

* Exclusive dosage of Piperacillin.

**Table 3:** Pharmacokinetic characteristics of the antibiotics under examination (values in means and standard deviations in brackets).

Antibiotics	AUC	Ke^−1h^	CL_tot_ (L/h)	CL_CVVH_ (ml/min)	V_d_ (L/Kg)
Vancomycin	453.1 (109.23)	0.04 (0.003)	1.8 (0.6)	34.7 (13.5)	0.54 (0.15)
Piperacillin	595.3 (413.8)	0.31 (0.08)	5.7 (1.4)	23.7 (6.06)	0.26 (0.07)
Ciprofloxacin	23.1 (15.5)	0.18 (0.03)	19.1 (8.7)	29.2 (11.9)	1.39 (0.47)
Meropenem	205.1 (124.9)	0.26 (0.06)	7.7 (4.8)	29.4 (10.6)	0.33 (0.18)
Levofloxacin	156.9 (77.2)	0.07 (0.0009)	3.6 (1.2)	27.4 (11.5)	0.6 (0.16)
Linezolid	105.5 (42.5)	0.18 (0.02)	7.6 (6.4)	26.09 (7.92)	0.51 (0.31)

**Table 4:** Antibiotic concentration lost with ultrafiltration.

Antibiotics	Lost with Ultrafiltrate mg/die	
	Mean (SD)	Median (IQR)
Vancomycin	32.6 (10.9)	33.3 (28–36)
Ciprofloxacin	6.6 (2.8)	6.04 (4.5–7.1)
Levofloxacin	16.8 (3.4)	18.29 (16.2–22.4)
Piperacillin	248.7 (128.6)	193.5 (182.3–205.4)
Meropenem	76.4 (34.4)	78.4 (69.7–86.7)
Linezolid	27.4 (17.6)	27 (24–33)
**Antibiotics**	**Lost with Ultrafiltrate mg/h**	
	Mean (SD)	Median (IQR)
Vancomycin	783 (262.7)	799.2 (680.1–875.6)
Ciprofloxacin	159.4 (67.8)	145.2 (134.5–166.3)
Levofloxacin	404.9 (82.4)	38.96 (34.7–45.8)
Piperacillin	5979.6 (3086.8)	4644 (3982–5141)
Meropenem	1833.4 (824.9)	1883 (1587–2057)
Linezolid	658.6 (422.4)	648 (578–658))
**Antibiotics**	**Daily dose mg/die**	
	Mean (SD)	Median (IQR)
Vancomycin	1518.2 (337.1)	1500 (1250–1750)
Ciprofloxacin	600 (282.8)	600 (400–600)
Levofloxacin	500 (0)	500 (/)
Piperacillin	11,250 (3765)	9000 (/)
Meropenem	3222.2 (1093)	3000 (/)
Linezolid	1200 (379.5)	1200 (/)

**Table 5:** Methods for estimating antibiotic dosage in CRRT [17].

Authors	CRRT Type	Equation	Assumption
Golper et al.	CVVH	D = C_ss_ × UBF × UFR × I	Dosage of antibiotic concentrations Sieving coefficient corresponding to the unbound fraction of the drug
Bugge et al.	CVVHDF	$D=D_{N}\left(P_{x}+\left(1-P_{x}\right)\frac{{\mathrm{Cl}}_{\mathrm{CRtot}}}{{\mathrm{Cl}}_{\mathrm{CRn}}}\right)$	Dosage of antibiotic concentrations Sieving coefficient corresponding to the unbound fraction of the drug The normal dose of the drug is sufficient for optimal action
Schetz et al.	CVVH	$D=D_{N}$	The normal dose of the drug is sufficient for optimal action
Schetz et al.	Tutte	$D=\frac{D_{\mathrm{anuria}}}{1-\left(\frac{{\mathrm{Cl}}_{\mathrm{EC}}}{{\mathrm{Cl}}_{\mathrm{EC}}+{\mathrm{Cl}}_{\mathrm{NR}}+{\mathrm{Cl}}_{R}}\right)}$	Dosage of antibiotic concentrations The drug dose in anuric patients is sufficient for optimal action

C_ss_: blood concentration at steady-state; Cl_anur_: clearance of the drug in anurics; Cl_CRn_: normal creatinine clearance; Cl_Rtot_: renal and extracorporeal clearance; Cl_EC_: extracorporeal clearance; Cl_N_: normal total clearance; Cl_NR_: non-renal clearance; Cl_R_ renal clearance; D_anuria_: recommended dose in anuric patients; D*_N_*: recommended dose in patients with normal renal function; I: interval between doses; P_x_ = fraction of extrarenal clearance (=Cl_anur_/Cl_N_); C_s_: Sieving coefficient; UBF: unbound fraction; UFR: ultrafiltration rate.

**Figure A1:**
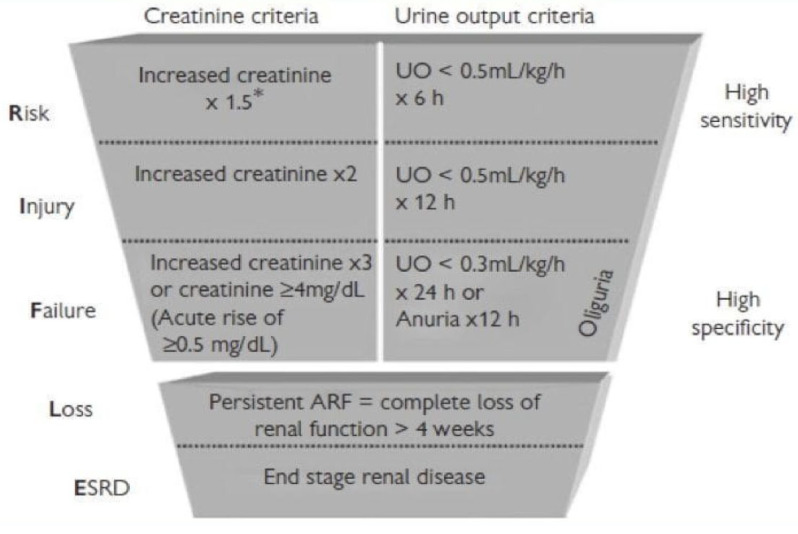
AKIN versus RIFLE CRITERIA. * times.

## Data Availability

Not applicable.

## Abbreviations

UF	ultrafiltrate
AKI	Acute kidney failure
ICU	intensive care unit
CRRT	continuous renal replacement therapy
V_d_	Volume of distribution of the drug (antimicrobial)
T_1/2_	half life of the drug
SMR	Standardised Mortality Ratio
AUC	area under the curve
Ke^−1h^	elimination rate constant in the first hour (of the drug)
CL_tot_ L/h	total clearance of the drug
ANOVA	Analysis of Variance

## Appendix A

**Table A1:** As far as staging is concerned, KDIGO identifies three levels.

Stage	Creatinine	Urinary Output
1	1.5–1.9 higher than the baseline levels or increasing >0.3 mg/dL	<0.5 mL/kg/h for 6–12 h
2	2–2.9 higher than the baseline levels	<0.5 mL/kg/h for >12 h
3	3 higher than the baseline levels or increasing >4 mg/dL or CRRT or reduction of eGFR <35 mL/min/1.73 m^2^ in pts <18 yrs.	<0.3 mL/kg/h for >24 or anuria for >12 h

**Table A2:** Classification of AKI proposed by AKIN.

STAGE	S-CREATININE CRITERIA	URINE OUTPUT CRITERIA
1	S-Creatinine increased >0.3 mg/dL or >150–200% from baseline	<0.5 mL/kg/h for 6 h
2	S-Creatinine increased >200–300% from baseline	<0.5 mL/kg/h for 12 h
3	S-Creatinine increased >300% from baseline or S-Creatinine >4 mg/dL with an acute increase of at least 0.5 mg/dl	<0.3 mL/kg/h for 24 h or Anuria for 12 h
